# Lysosomal Trafficking of TGFBIp via Caveolae-Mediated Endocytosis

**DOI:** 10.1371/journal.pone.0119561

**Published:** 2015-04-08

**Authors:** Seung-il Choi, Yong-Sun Maeng, Tae-im Kim, Yangsin Lee, Yong-Sun Kim, Eung Kweon Kim

**Affiliations:** 1 Department of Ophthalmology, Corneal Dystrophy Research Institute, Yonsei University College of Medicine, Seoul, South Korea; 2 Institute of Vision Research, Severance Biomedical Science Institute, Brain Korea 21 Plus Project for Medical Science, Yonsei University College of Medicine, Seoul, South Korea; 3 Department of Integrated Omics for Biomedical Science, Graduate School, Yonsei University, Seoul, South Korea; 4 Ilsong Institute of Life Science, Hallym University, Anyang, South Korea; 5 Department of Microbiology, College of Medicine, Hallym University, Chuncheon, South Korea; The Chinese University of Hong Kong, HONG KONG

## Abstract

Transforming growth factor-beta-induced protein (TGFBIp) is ubiquitously expressed in the extracellular matrix (ECM) of various tissues and cell lines. Progressive accumulation of mutant TGFBIp is directly involved in the pathogenesis of TGFBI-linked corneal dystrophy. Recent studies reported that mutant TGFBIp accumulates in cells; however, the trafficking of TGFBIp is poorly understood. Therefore, we investigated TGFBIp trafficking to determine the route of its internalization and secretion and to elucidate its roles in the pathogenesis of granular corneal dystrophy type 2 (GCD2). Our data indicate that newly synthesized TGFBIp was secreted via the endoplasmic reticulum/Golgi-dependent secretory pathway, and this secretion was delayed in the corneal fibroblasts of patients with GCD2. We also found that TGFBIp was internalized by caveolae-mediated endocytosis, and the internalized TGFBIp accumulated after treatment with bafilomycin A1, an inhibitor of lysosomal degradation. In addition, the proteasome inhibitor MG132 inhibits the endocytosis of TGFBIp. Co-immunoprecipitation revealed that TGFBIp interacted with integrin α_V_β_3_. Moreover, treatment with arginine-glycine-aspartic acid (RGD) tripeptide suppressed the internalization of TGFBIp. These insights on TGFBIp trafficking could lead to the identification of novel targets and the development of new therapies for TGFBI-linked corneal dystrophy.

## Introduction

TGFBI-linked corneal dystrophies are autosomal dominant disorders caused by mutations in transforming growth factor-beta-induced (*TGFBI*) gene [1–3]. These disorders are characterized by age-dependent progressive accumulation of deposits of mutant TGFBIp in the corneal epithelia and stroma, followed by interference with corneal transparency [1]. Granular corneal dystrophy type 2 (GCD2) is an autosomal dominant disorder caused by a mutation in codon 124 of the *TGFBI* gene that results in an arginine-to-histidine substitution (R124H) [1].

TGFBIp is expressed in various tissues such as cornea, skin, lung, bone, bladder, and kidney [4,5]. TGFBIp is an extracellular matrix (ECM) protein with a secretory signal sequence and cysteine-rich (EMI) domain at the N-terminus, four homologous internal fasciclin (FAS1) domains, and an Arg-Gly-Asp (RGD) integrin recognition sequence at the C-terminus [6]. *In vitro* studies have shown that TGFBIp mediates cell growth [7], cell differentiation [8], wound healing [9], cell adhesion [10], migration [11], apoptosis [12], proliferation [11], and tumorigenesis [13]. Moreover, TGFBIp mediates migration and cell adhesion through its interaction with cell surface integrin receptors [14–16].

Most secretory proteins contain signal peptides that lead to direct sorting to the endoplasmic reticulum (ER). These proteins are typically trafficked to the plasma membrane or ECM through the ER/Golgi secretory pathway [17], although some proteins are trafficked via an unconventional non-ER/Golgi secretory pathway. After ER translocation, proteins are packaged into coated vesicles that either fuse directly with the plasma membrane or with endosomal or lysosomal compartments before adhering to the plasma membrane. Alternatively, proteins can be packaged into non-coated vesicles that fuse directly with the plasma membrane or are targeted to the Golgi apparatus before reaching the ECM [18]. In addition to its recognition as a cellular degradation pathway that delivers cytoplasmic proteins and organelles to lysosomes for subsequent degradation, autophagy has also been demonstrated to play a role in unconventional protein secretion [19].

Endocytosis is a basic cellular process in eukaryotic cells that leads to the internalization of molecules from the cell surface. Internalized molecules from the plasma membrane are recycled back to the surface or sorted to lysosomes for degradation. Endocytosis could be classified into two broad categories: phagocytosis (the internalization of large particles) and pinocytosis (the internalization of fluids and solutes) [20]. The major endocytic pathways are usually distinguished by their differential sensitivity to inhibitors [21]; for example, caveolae- and lipid raft-mediated endocytosis from the clathrin-dependent pathway can be identified by sensitivity to non-acute cholesterol depletion with agents such as filipin, genistein, nystatin, or methyl-β-cyclodextrin.

Degradation and removal of ECM proteins is associated with several physiological processes, including tissue development, remodeling, and repair [5,22]. ECM remodeling is controlled by matrix synthesis, deposition, and degradation. Two molecular mechanisms are believed to be involved in ECM turnover. The first pertains to extracellular degradation of ECM proteins by matrix metalloproteases and other proteases [23,24], and the second involves lysosomal degradation of internalized ECM proteins following endocytosis [22,25,26]. Impaired ECM homeostasis contributes to the progression of many diseases, including fibrosis, arthritis, and cancer [27–31].

Recently, we demonstrated that mutation in *TGFBI* causes aberrant redistribution of TGFBIp into lysosomes [32]. Mutant TGFBIp also accumulated in lysosomal compartments as a result of defective autophagy [33]. In this study, we sought to gain a better understanding of the molecular events involved in the trafficking and turnover of ECM proteins containing TGFBIp. Specifically, we investigated the intracellular and extracellular trafficking of TGFBIp and its involvement in the pathogenesis of TGFBI-linked corneal dystrophy. Our data demonstrate that TGFBIp secretion occurs via the ER/Golgi-dependent secretory pathway. However, this process is delayed in GCD2 corneal fibroblasts. Furthermore, we discovered that TGFBIp is internalized via a caveolin-dependent integrin-mediated endocytic pathway and is trafficked directly to the lysosomes. The findings of this study will enable the identification of therapeutic targets for the treatment of TGFBI-linked corneal dystrophy.

## Materials and Methods

### Materials

Chlorpromazine, nystatin, genistein, MG132, bafilomycin A1 (Baf-A_1_), monensin (MON), brefeldin A (BFA), cycloheximide (CHX), Arg-Gly-Asp (RGD)-containing peptide (Gly-Arg-Gly-Asp-Ser-Pro [GRGDSP]), and RAD control peptide (Gly-Arg-Ala-Asp-Ser-Pro [GRADSP]) were obtained from Sigma-Aldrich (St Louis, MO, USA). hTERT-inducible lentiviral particles were obtained from GenTarget, Inc. (San Diego, CA, USA). The cell lines HEK293T (ATCC CRL-3216), NIH3T3 (ATCC CRL-1658), SK-N-MC SK-N-SH (ATCC HTB-11), and 3T3 MEF KO (ATCC CRL-2753) was purchased from the American Type Culture Collection (ATCC) (Rockville, MD).

### Ethics statement

This study was carried out according to the tenets of the Declaration of Helsinki and it followed international ethic requirements for human tissues. The Severance Hospital Institutional Review Board (IRB: 4-2010-0013) at Yonsei University approved use of corneal biopsy specimens of patients and controls for study of pathological mechanisms of GCD2 and has previously been described [34]. All participants provided written informed consent to participate in this study. The study protocol was approved by the Severance Hospital IRB (CR04124) at Yonsei University. Written informed consent from participating donors and informed assent from were all participants obtained according to institution’s IRB policies.

### Cell culture

All the cells were cultured in Dulbecco’s modified Eagle’s medium (DMEM; Corning Cellgro, Manassas, VA, USA) supplemented with 10% fetal bovine serum (FBS; Gibco, Carlsbad, CA, USA), 100 IU/ml penicillin (Corning Cellgro), 100 μg/mL streptomycin (Corning Cellgro), and phosphate-buffered saline (PBS; Corning Cellgro). The cells were maintained in a 5% CO_2_ incubator at 37°C.

### Pulse-chase metabolic labeling and immunoprecipitation

Normal and GCD2 corneal fibroblasts were plated onto 35-mm culture dishes and cultured for 24 h. Cells were pre-incubated with cysteine-free DMEM (Sigma-Aldrich) containing dialyzed 0.5% fetal bovine serum (Invitrogen). Cells were incubated (pulsed) with 0.3 mCi/mL [^35^S] cysteine (PerkinElmer Life and Analytical Sciences, Boston, MA, USA) in the same medium for 20 min. After labeling, the cells were washed three times with PBS and incubated (chased) with DMEM containing 10% FBS at 37°C for 0, 15, 30, 60, 120, 180, or 240 min. The medium was collected and centrifuged at 4,500 × *g* for 10 min at 4°C. The cells were harvested in PBS, lysed in 500 μL RIPA buffer containing protease inhibitors, and centrifuged at 10,000 × *g* for 10 min at 4°C. Cell lysates and media were each divided into two equal volumes and immunoprecipitated with anti-TGFBIp and Dynabeads coated with sheep anti-mouse IgG (Invitrogen Dynal). The immunoprecipitated proteins were separated by 10% Tris/Glycine SDS-PAGE. The gels were enhanced with ENHANCE (Amersham Pharmacia Biotech) and exposed to Kodak BioMax X-ray films (Kodak, Rochester, NY, USA) for 7 days at −80°C.

### Internalization assay and endocytic inhibitors

TGFBIp internalization assays were performed at 37°C and 4°C with or without the following endocytic inhibitors: 10–100 μg/mL chlorpromazine, 100 μg/mL genistein, and 25 μg/mL nystatin. The involvement of integrins in TGFBIp internalization was examined by incubating corneal fibroblasts with an integrin peptide inhibitor (1–10 μM RGD peptides) in the presence of ~1 μg/mL TGFBIp. Corneal fibroblasts were incubated for 60 min in a medium containing CHX to block endogenous TGFBIp synthesis and/or with the inhibitors of endocytosis. The specificity of each treatment was confirmed by incubating the cells with exogenous TGFBIp at 4°C, to allow binding, before incubation at *37*°*C* for 120 min, to allow endocytosis. After incubation, the medium was removed and the cells were washed three times with cold PBS. Endocytosis of TGFBIp was analyzed by western blotting and immunofluorescence staining.

### Isolation, immortalization, and culture of primary corneal fibroblasts

Normal and GCD homozygous primary corneal fibroblasts were prepared using previously described methods [34]. GCD2 was diagnosed by DNA sequence analysis for *TGFBI* gene mutations. The normal human corneal fibroblast cell line [35] was kindly provided by Dr. James Jester. GCD2 corneal fibroblasts were immortalized by expression of the catalytic subunit of human telomerase (hTERT) using a reversible retroviral expression vector [36]. The primary corneal fibroblasts (Table 1) were cultured in DMEM supplemented with 10% fetal bovine serum at 37°C in a humidified incubator with 95% air and 5% CO_2_. All the cell lines were cultured in DMEM with 10% fetal bovine serum at 37°C in a humidified incubator with 95% air and 5% CO_2_.

**Table 1:** Cases used for analysis in this Study.

Case No.	Pathological diagnosis	Gender	Age	Mean age
1	CON-WT	F	20	
2	CON-WT	M	10	26.25
3	CON-WT	M	29	
4	CON-WT	M	46	
5	GCD2-HE	F	37	
6	GCD2-HE	F	20	27
7	GCD2-HE	M	24	
8	GCD2-HO	F	27	
9	GCD2-HO	M	10	
10	GCD2-HO	F	13	18
11	GCD2-HO	M	22	

CON-WT, control case; GCD2-HE, heterozygote of granular corneal dystrophy type 2 case; GCD2-HO, homozygote of granular corneal dystrophy type 2 case; F, female; M, male.

### Preparation of cell lysates, co-immunoprecipitation, and western blot analysis

Cell lysates from corneal fibroblasts were prepared in a radio-immunoprecipitation assay buffer (RIPA buffer; 150 mM NaCl, 1% NP-40, 0.5% deoxycholate, 0.1% SDS, 50 mM Tris-HCl, pH 7.4) containing protease inhibitors (Complete Mini Protease Inhibitor Cocktail Tablet, Roche Applied Science, Indianapolis, IN, USA) and phosphatase inhibitors (PhosSTOP, Roche Applied Science). Crude cell lysates were centrifuged at 10,000 × *g* for 10 min at 4°C to remove nuclear fragments and tissue debris. A portion of the supernatant was used to determine the total protein concentration with a bicinchoninic acid kit (Pierce). Total cellular protein was electrophoresed on Tris-glycine SDS polyacrylamide gels. Proteins were transferred onto polyvinylidene difluoride (PVDF) membranes (Millipore Corp., Bedford, MA, USA), blocked in 5% dry milk in Tris-buffered saline containing Tween-20 (TBS-T; 20 mM Tris, 150 mM NaCl, pH 7.5 containing 0.1% Tween 20) at room temperature for 1 *h*, washed three times with TBS-T, and then incubated with primary antibodies to TGFBIp (0.2 μg/mL; R&D Systems, Minneapolis, MN, USA), caveolin-1 (BD Biosciences, San Jose, CA, USA), caveolin-2 (BD Biosciences), and β-actin (Sigma-Aldrich) overnight at 4°C. After three washes with TBS-T, the blots were incubated at room temperature for 1 h with horseradish peroxidase-conjugated anti-mouse IgG or anti-rabbit IgG secondary antibody (Amersham Pharmacia Biotechnology, Piscataway, NJ). Western blots were visualized using the Super Signal West Pico Chemiluminescent Substrate (Pierce). Immunoreactive protein bands were scanned at two intensities, and the optical densities of the bands were quantified using the ImageJ software (version 1.37, Wayne Rasband, NIH, Bethesda, MD), corrected by background subtraction, and normalized to the intensity of the corresponding β-actin protein bands.

Co-immunoprecipitation was performed with magnetic beads coated with polyclonal anti-integrin α_V_β_3_ (Chemicon, Temecula, CA, USA) and α_V_ antibody (Chemicon), according to the manufacturer’s instructions (Invitrogen Dynal, Carlsbad, CA, USA). Immunoprecipitated proteins were separated by SDS-PAGE (8−16% gradient Tris-glycine gel; Komabiotech, Seoul, Korea) and immunoblotted with TGFBIp antibody (0.2 μg/mL; R&D Systems).

### Immunocytochemical staining

WT and GCD2 homozygous mutant (HO) corneal fibroblasts were grown on culture slides (Cat. No. REF 354108; BD Falcon, Labware, Franklin Lakes, NJ, USA), permeabilized, and fixed in methanol at −20°C for 3 min. Cells were washed in PBS, blocked with 10% bovine serum albumin (Sigma-Aldrich) in PBS for 10 min, and incubated sequentially with primary antibodies in blocking buffer at 4°C overnight, followed by secondary antibodies for 1 h at room temperature. Coverslips were mounted on glass slides with Vectashield Mounting Medium (Vector Labs Inc., Burlingame, CA, USA) and viewed under a Leica TCS SP5 confocal microscope (Leica Microsystems, Wetzlar, Germany). The following primary antibodies were used: monoclonal anti-TGFBIp (kindly provided by Dr. I S Kim, Kyungpook National University, Korea), polyclonal anti-trans-Golgi network (TGN) 38 (Santa Cruz Biotechnology, Santa Cruz, CA), anti-GM130 (Cell Signaling Technology, Beverly, MA), anti-mannosidase II (Abcam, Cambridge, UK), and anti-Cathepsin D rabbit polyclonal antibody (Calbiochem, La Jolla, CA, USA). Alexa 594-conjugated cholera toxin B subunit (CTxB) was obtained from Molecular Probes, Inc. (Eugene, Oregon, USA). The following secondary antibodies were used: Alexa 594 (red)-conjugated anti-rabbit IgG (Vector Laboratories Inc., DI-1488) and fluorescein isothiocyanate (green)-labeled anti-mouse IgG (Jackson ImmunoResearch Laboratories, West Grove, PA).

### Transmission electron microscopy

Cultured corneal fibroblasts were fixed overnight, dehydrated, and processed for electron microscopy analysis as described previously [37]. Evaluation was performed using a transmission electron microscope (JEM1200 EX2; JEOL Ltd.). For immunogold-labelling experiments, the primary antibody (monoclonal anti-TGFBIp antibody) was detected via the standard protein A–gold method [38].

### Statistical analysis

Data were evaluated statistically to determine significant differences (P *<* 0.05) using one-way analysis of variance (ANOVA), followed by the Newman−Keuls multiple comparison test. Data are expressed as mean ± SD. All the data were processed using the scientific graphing analysis software (Prism, version 5.0; GraphPad Software Inc., San Diego, CA, USA).

## Results

### Secretion of TGFBIp via an ER/Golgi-dependent pathway is delayed in GCD2 corneal fibroblasts

**Fig 1:**
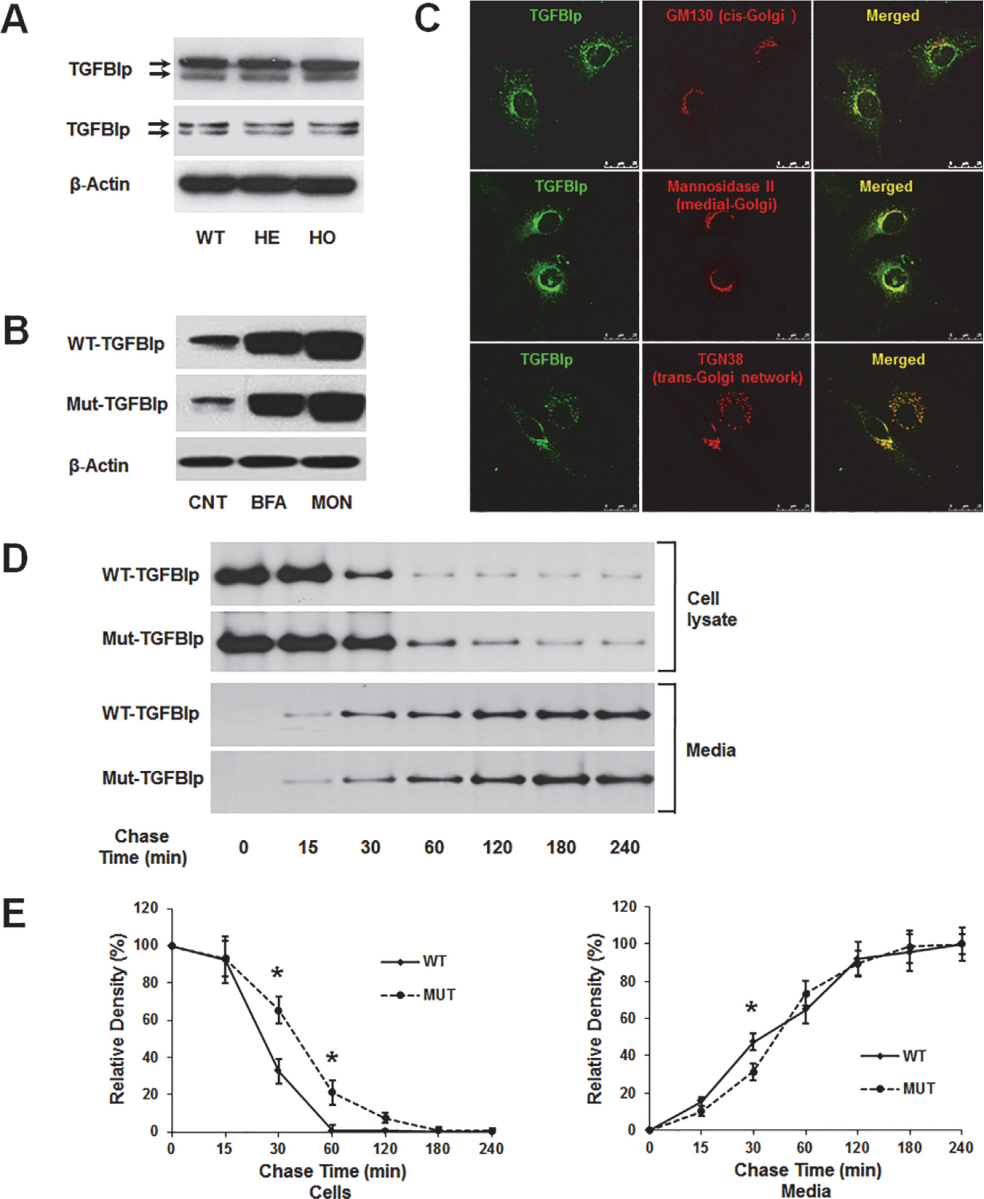
Expression and secretion of TGFBIp in wild-type (WT) and heterozygous (HE) or homozygous (HO) mutant primary corneal fibroblasts. **A**. Western blot analysis of TGFBIp in cell lysates (upper panel) and conditioned media (lower panel) of WT, HE, and HO cells. β-actin was used as a loading control. Molecular weight markers (in kDa) are indicated. **B**. Secretion of WT and mutant TGFBIp was inhibited by treatment with brefeldin A (BFA) and monensin (MON). **C**. TGFBIp co-localized with markers of cis-Golgi (upper panel), medial-Golgi (middle panel), and trans-Golgi (lower panel) in cortical cells. Representative confocal images of immunofluorescence staining of TGFBIp (green) with GM130, mannosidase II, and TGN38 (all red) are shown. Overlapping areas are displayed in yellow in the merged images. Bars = 25 μm. **D**. Secretion of mutant TGFBIp is delayed in GCD2 corneal fibroblasts. Corneal fibroblasts from a patient with a homozygous TGFBIp mutation and a WT control were pulse-labeled for 20 min using ^35^S-cysteine and then incubated for 0, 15, 30, 60, 120, 180, and 240 min in unlabeled media before immunoprecipitation of TGFBIp from cell lysates and conditioned media. Phosphorimaging was performed after SDS-PAGE to detect TGFBIp. One representative experiment is shown. **E**. Quantitation of the experiment presented in **D**. Triplicate lysate samples were analyzed. *P < 0.05.

Although studies have established that TGFBIp protein is secreted into the ECM and degraded via autophagy/lysosomal pathway, the underlying trafficking pathway(s) remain poorly understood. To address this issue, we examined the secretion of TGFBIp in the presence of brefaldin-A (BFA), which reversibly blocks protein transport from the *ER* [39] and monensin (MON), which effectively inhibits protein transport from medial to trans Golgi cisternae [40,41]. In normal culture conditions, western blot analysis of TGFBIp in cell lysates and conditioned media of wild-type (WT) and GCD2 homozygous (HO) primary fibroblasts indicated two forms of the protein at ~66 and ~68 kDa (Fig 1A). The effects of inhibitors on TGFBIp secretion were assessed by incubating cells for 6 h at 37°C in a media containing 5 μg/mL BFA or 5 μM MON. Cells were lysed and western blot analysis of TGFBIp in cell lysates was performed. As shown in Fig 1, TGFBIp secretion was nearly completely abrogated by exposure to BFA and MON (Fig 1B) in both WT and GCD2 HO corneal fibroblasts. These data demonstrate that TGFBIp is transported from the ER lumen to the Golgi apparatus and then to the ECM via the ER/Golgi-dependent secretory pathway; also termed the classical or conventional secretory pathways. In addition, our data demonstrate that TGFBIp co-localized with the Golgi proteins GM130 (a cis-Golgi matrix protein), mannosidase II (a medial-Golgi enzyme), and TGN38 (trans-Golgi network protein 38), suggesting the secretion of newly synthesized TGFBIp into the ECM via the ER/Golgi-dependent secretory pathway (Fig 1C).

Studies have demonstrated intracellular accumulation of mutant TGFBIp in GCD2 corneal fibroblasts [33], suggesting the inhibition of TGFBIp secretion. To investigate this possibility, we performed a pulse-chase experiment in combination with immunoprecipitation to dynamically monitor the secretion of newly synthesized TGFBIp in WT and GCD2 HO corneal fibroblasts. Corneal fibroblasts were pulse-labeled with ^35^S-cysteine for 20 min and then chased for different time periods. Protein extracts were harvested and subjected to immunoprecipitation with an anti-TGFBIp monoclonal antibody, followed by 10% SDS-PAGE and autoradiography. The amounts of secreted and intracellular TGFBIp were quantitated by densitometry. Our results clearly showed that WT and mutant TGFBIp exhibited different trafficking kinetics (Fig 1D and 1E). At 60 min, nearly all of the newly synthesized WT TGFBIp was secreted into the culture media, with only 0.5% (standard deviation [SD] ± 0.9%) remaining inside the cells, whereas 20% (± 0.6%) of mutant TGFBIp was retained inside the cells (Fig 1D and 1E). Most of the mutant TGFBIp was not secreted into the culture media until 180 min after the chase (when only 7 ± 4.6% remained inside the cells). Moreover, in normal corneal fibroblasts, the cellular TGFBIp level decreased to approximately 50% at 20 min (± 2 min) post chase, whereas a similar reduction in GCD2 homozygous corneal fibroblasts expressing mutant TGFBIp was observed after 45 min (± 4 min) post chase.

### TGFBIp was internalized by endocytosis

**Fig 2:**
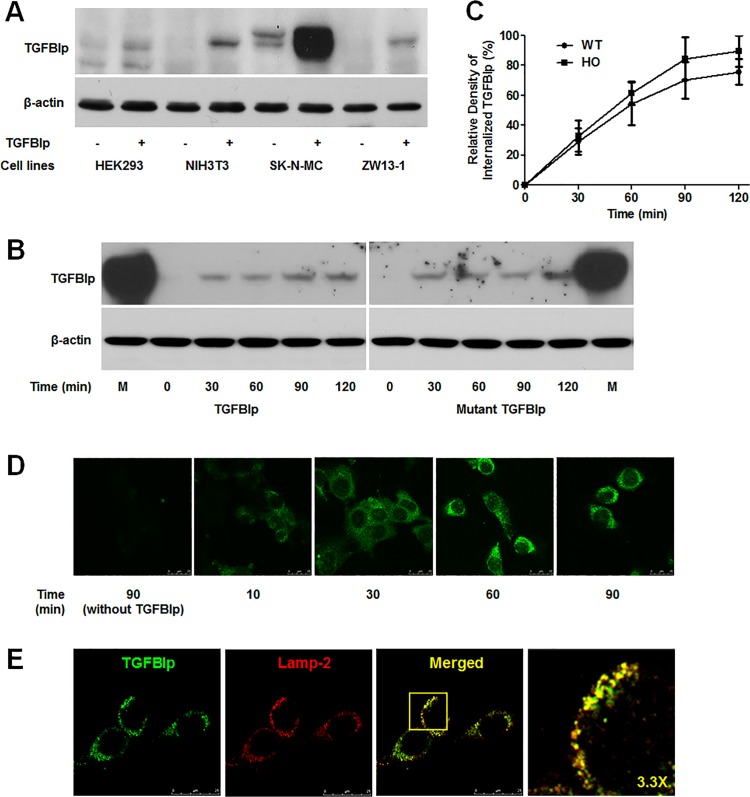
Internalization of TGFBIp in various cell lines. **A**. TGFBIp was internalized in NIH3T3, SK-N-MC, and ZW13-1 cell lines but not in HEK293T. Cells were pre-incubated at 4°C for 30 min in basal medium, and then incubated for a further 120 min at 37°C in basal or TGFBIp-supplemented (~1 μg/mL) medium. Cells were washed twice with cold PBS on ice, and surface-bound TGFBIp was removed by washing three times with ice-cold acidic buffer. Cells were harvested by scraping into ice-cold PBS, pelleted by centrifugation at 1,000 × *g*, lysed in RIPA buffer, and 50 μg of lysate was used for western blot analysis. **B**. Internalization of mutant TGFBIp and WT TGFBIp was similar in NIH3T3 cells. Cells were treated as in **A** and analyzed by western blotting. **C**. Internalization of WT- and Mut-TGFBIp in the NIH3T3 cell line. Cells were incubated at 4°C for 30 min and were then shifted to 37°C in the continuous presence of TGFBIp. At the indicated time points, cells were analyzed for the amount of internalized TGFBIp. The experiment was repeated three times independently. There were no statistically significant differences between the rates of WT- and Mut-TGFBIp internalization (p > 0.05). **D**. Visualization of TGFBIp internalization in NIH3T3 cells by confocal microscopy. Cells were grown on glass coverslips and treated as in **A**, before fixation in methanol at −20°C for 3 min. Immunocytochemical staining was performed with monoclonal anti-TGFBI antibody, as described in Materials and Methods. **E**. TGFBIp co-localizes with Lamp-2. NIH3T3 cells grown on glass slides were subjected to immunocytochemical staining with monoclonal anti-TGFBIp and anti-Lamp-2 antibodies as described in Materials and Methods. Coverslips were mounted on the glass slides with mounting medium, and the cells were viewed using a Leica TCS SP5 confocal microscope.

In spite of its extensive distribution throughout the ECM, lysosomal degradation of TGFBIp suggests the possibility of TGFBIp internalization through endocytosis. Therefore, we investigated the endocytosis of TGFBIp in four cell lines: HEK293T, NIH3T3, ZW13-1 [42], and SK-N-MC. The cells were incubated with exogenous TGFBIp for 2 h at 37°C to allow internalization. Quantification of the results showed that HEK293T cells, which do not express TGFBIp, did not internalize the protein. However, NIH3T3 and ZW13-1 cells, which also do not express TGFBIp, clearly demonstrated TGFBIp internalization. Furthermore, we detected a higher level of internalized TGFBIp in SK-N-MC cells, which endogenously express TGFBIp (Fig 2A).

We also analyzed time-dependent TGFBIp internalization by western blot (Fig 2B and 2C) and immunocytochemical staining (Fig 2D). The results showed that TGFBIp internalization into the cells started immediately after warming, and proceeded rapidly until reaching a maximum (89% ± 17% and 98% ± 20% in WT- and Mut-TGFBIp, respectively) within 90 min (Fig 2C). After warming, the uptake ratio of TGFBIp internalization reached 29% ± 12% and 54% ± 20% in WT-TGFBIp, and 32% ± 14% and 61% ± 10% in Mut-TGFBIp at 30 min and 60 min, respectively. However, the difference in the uptake ratio was not significant between WT- and mut-TGFBIp (Fig 2C).

Confocal microscopy revealed TGFBIp internalization after incubation for only 10 min (short incubation period) (Fig 2D). Longer incubation times (60–90 min) led to localization of TGFBIp in the central part of the cells. Finally, staining for Lamp-2, a marker of late endosomes/lysosomes, revealed co-localization with TGFBIp, suggesting that TGFBIp was trafficked to lysosomes in the NIH3T3 cell line (Fig 2E).

### Internalization of TGFBIp occurs via caveolae-mediated endocytosis

**Fig 3:**
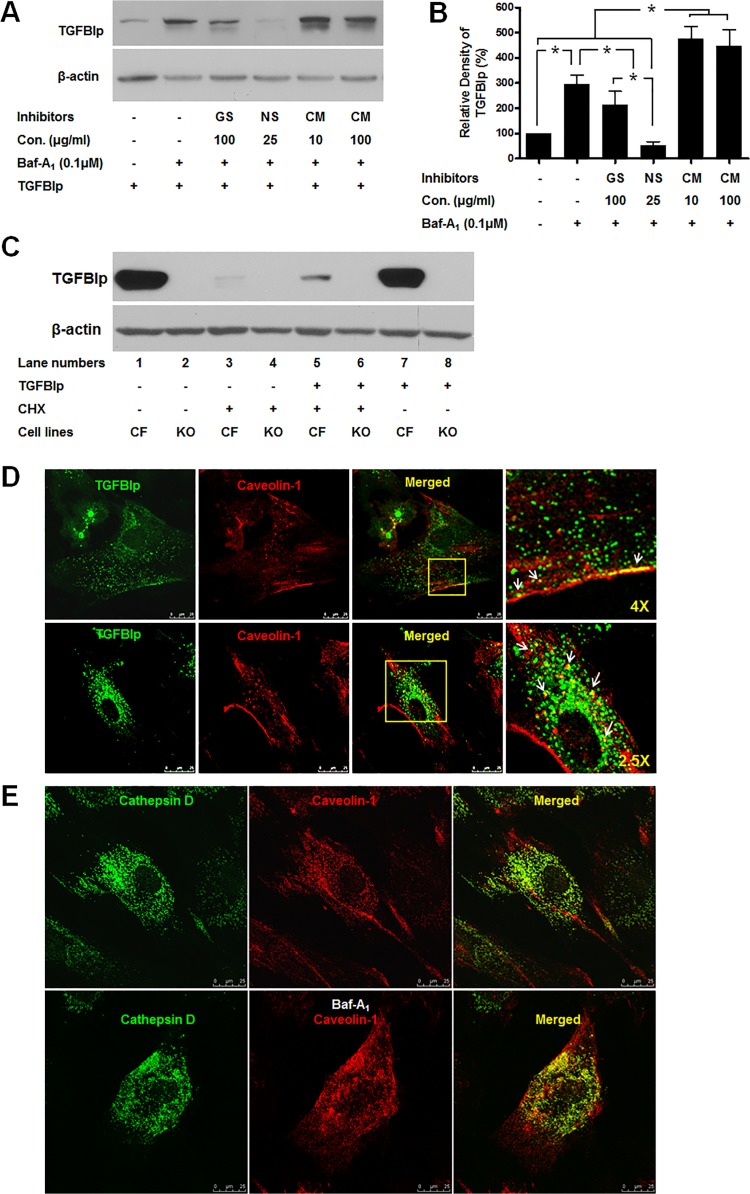
Effect of inhibitors of caveolae- and clathrin-dependent endocytosis on TGFBIp internalization. **A**. Inhibitors of endocytosis decreased TGFBIp levels in corneal fibroblasts. Cells were left untreated (lane 1) or treated with Baf-A_1_ and various endocytosis inhibitors for 60 min before incubation with TGFBIp for 120 min. TGFBIp levels were determined by western blot analysis. GS; genistein, NS; nystatin, CM; chlorpromazine. Data from one representative experiment is shown. β-actin was used as a loading control. **B**. Densitometric quantitation of the experiment presented in **A**. Data represent the TGFBIp/β-actin ratio and expressed as mean ± SD of three independently treated samples from one or two experiments. ANOVA analysis of TGFBIp levels across the treatment conditions showed no significant changes. *P≤0.05 relative to controls by Student’s *t*-test. **C**. Internalization of TGFBIp in WT corneal fibroblasts and caveolin-1-null cell line (3T3 MEF CAV-1 KO). 3T3 MEF CAV-1 KO cell does not express TGFBIp (lane 2 and 4). TGFBIp was internalized in corneal fibroblasts (lane 5) but not in 3T3 MEF CAV-1 KO cell lines (lanes 6 and 8). Cells were pre-incubated at 37°C for 60 min in basal medium or basal medium with CHX, and then incubated for a further 60 min at 37°C in basal or TGFBIp-supplemented (~1 μg/mL) medium with or without CHX. Cells were washed twice with cold PBS on ice, and surface-bound TGFBIp was removed by washing three times with ice-cold acidic buffer. Cells were harvested by scraping into ice-cold PBS, pelleted by centrifugation at 1,000 × *g*, lysed in RIPA buffer, and 50 μg of total protein was used for western blot analysis. **D**. TGFBIp co-localizes with caveolin-1 in corneal fibroblasts. Cells were grown on glass slides for 12 h and then fixed using methanol at −20°C and incubated with antibodies against TGFBIp and caveolin-1. Localization of TGFBIp (green) and caveolin-1 (red) are shown. Areas of TGFBIp and caveolin-1 co-localization appear as yellow regions in the merged image. The boxed area in the third panel was magnified and is presented as the fourth panel. Arrows in the fourth panel identify regions of TGFBIp and caveolin-1 co-localization. **E**. Cathepsin D co-localizes with caveolin-1 in corneal fibroblasts in the absence and presence of Baf-A_1_. Cells were grown on glass slides for 12 h in the absence (upper panel) or presence (lower panel) of Baf-A_1_ (0.1 μM) and then fixed with methanol at −20°C and incubated with antibodies against cathepsin D and caveolin-1. Localization of cathepsin D (green) and caveolin-1 (red) is shown. Areas of cathepsin D and caveolin-1 co-localization appear as yellow regions in the merged image.

Next, we investigated whether TGFBIp internalization occurred via clathrin- or caveolae-mediated endocytosis. We treated a corneal fibroblast cell line with the general protein tyrosine kinase inhibitor genistein, which prevents caveolae-mediated internalization [43–46]. Our results showed that treatment with genistein (100 μg/mL) significantly reduced the amount of intracellular TGFBIp (Fig 3A and 3B, lane 3). To further confirm the route of TGFBIp internalization, we treated the cells with cholesterol-chelating drug nystatin, which interferes with caveolae-mediated endocytosis [47–49], and found that nystatin (25 μg/mL) caused a significant reduction in intracellular TGFBIp levels (Fig 3A and 3B, lane 4). In contrast, an inhibitor of clathrin-mediated endocytosis, chlorpromazine (10 or 100 μg/mL), increased the level of intracellular TGFBIp (Fig 3A and 3B, lanes 5), even at higher concentrations (Fig 3A and 3B, lanes 6). To further confirm whether TGFBIp is internalized via a caveolea-mediated endocytosis, we used caveolin-1-null cell line (3T3 MEF CAV-1 KO). RT-PCR and western blotting showed that 3T3 MEFs CAV-1 KO cells, which do not express *TGFBI* gene (S1 Table and S1 Fig), did not internalize TGFBIp (Fig 3C). These results clearly demonstrated that TGFBIp is internalized by caveolea-dependent endocytosis.

We also demonstrated the co-localization of TGFBIp and caveolin-1 in primary cultured corneal fibroblasts (Fig 3D). Caveolin-1 staining was localized to the plasma membrane and other intracellular compartments. Co-localization of caveolin-1 with cathepsin D was also observed, and it increased in the presence of Baf-A_1_, an inhibitor of protein degradation that blocks the fusion of the endosome with the lysosome [50] (Fig 3E). These data suggest that caveolae containing TGFBIp is also targeted by lysosomes for degradation after internalization.

**Fig 4:**
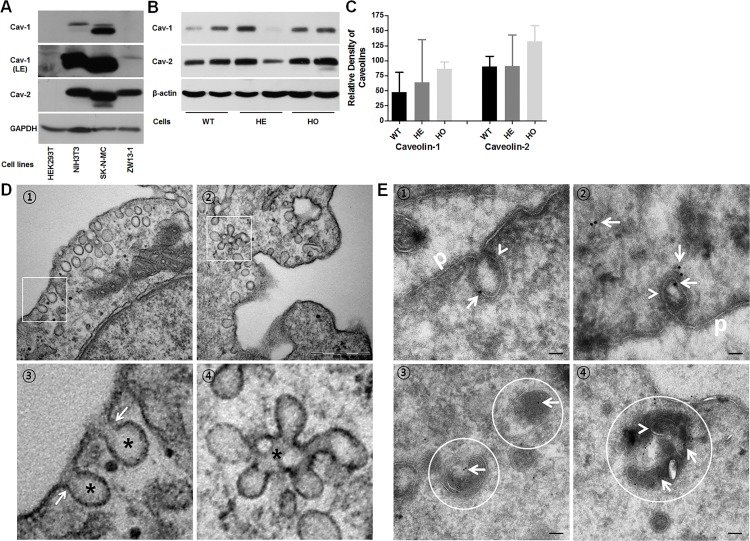
Expression of caveolin-1 and -2 in established cell lines and caveolae formation in WT, HE, and HO mutant TGFBIp-expressing primary cultured corneal fibroblasts. **A**. Total cellular protein (50 μg) from the specified cell lines was subjected to western blot analysis with anti-caveolin-1 (first panel), anti-caveolin-1 (second panel, LE: longer exposure), anti-caveolin-2 (third panel), and anti-Glyceraldehyde 3-phosphate dehydrogenase (GAPDH, fourth panel). GAPDH was used as a loading control. **B**. Western blot analysis of caveolin-1 and -2 expressions in WT, HE, and HO TGFBIp-expressing corneal fibroblasts. β-actin was used as a loading control. **C**. Densitometric quantitation of the experiment presented in **B**. **D**. Transmission electron microscopy (TEM) of corneal fibroblasts reveals formation of caveolae in corneal fibroblasts. Cells were grown in basal media, fixed, and then prepared for scanning TEM as described in Materials and Methods. ① Caveolae are detected on the inner surface of the plasma membrane of corneal fibroblasts. Cells contain caveolae in their apical membranes that are characterized by coat-free flask-shaped invaginations (asterisks in ③) with a diaphragm at the neck (arrows in ③). ② Note the characteristic clustering of caveolae into racemose structures, or caveosomes, on the basal side (④ asterisks). Bar = 500 nm. **E**. At steady state, TGFBIp is localized in the caveolae both at the plasma membrane and inside the cell. TGFBIp was immunogold-labeled on ultrathin cryosections of WT corneal fibroblasts using the TGFBIp monoclonal antibody. ① and ② plasma membrane caveolae-like structures (arrowhead) appeared as flask-shaped invaginations on the plasma membrane (p). ③ and ④ Ultrathin cryosections were labeled with anti-TGFBIp antibodies. TGFBIp-gold signal (arrow) accumulated in the lysosomes of WT corneal fibroblasts. ④ Caveolae-like structures also appeared in the lysosomes (circle). The scale bars in all panels are 100 nm.

Expression of exogenous caveolin-1 induces the formation of caveolae on the surface of cells that normally do not form them [51]. Furthermore, knockout of the gene encoding caveolin-1 in mice also reduces the expression of caveolin-2 and leads to the loss of morphologically defined caveolae [52]. These studies suggest a possible correlation between the expression of caveolins and the efficiency of TGFBIp endocytosis. Therefore, we examined the expression of caveolin-1 and -2 in cells that have different internalization levels of TGFBIp (Fig 4A). Caveolin-1 and -2 proteins showed cell-specific expression pattern (Fig 4A). Specifically, caveolin-1 was more abundant in SK-N-MC cells than in the NIH3T3 cells. Longer exposure of the blots showed a very low level of caveolin-1 expression in ZW13-1 cells. Caveolin-2 also showed differential expression with a moderate level of expression in ZW13-1 cells and a higher level in the NIH3T3 and SK-N-MC cell lines (Fig 4A). However, HEK293T cells had no detectable expression of caveolin-1 and caveolin-2 (Fig 4A), consistent with previously published data [53]. However, the low-level expressions of *CAV-1* were detected at mRNA level by RT-PCR (S1 Fig). Taken together, these results demonstrate that the expression of caveolin-1 protein closely resembled that of caveolin-2 and that there was a clear correlation between the cellular expression levels of caveolins and the endocytosis of TGFBIp in these cell lines. These results also suggest that the expression levels of caveolin-1 and -2 might influence TGFBIp endocytosis in corneal fibroblasts. Therefore, we analyzed the expression levels of caveolin-1 and -2 in WT and GCD2 corneal fibroblasts. Fig 3B shows that corneal fibroblasts differentially express caveolin-1 and -2, but the levels of expression did not differ significantly between WT and GCD2 corneal fibroblasts (Fig 4B and 4C).

To confirm the presence of caveolae and caveolar vesicles (caveosomes) in corneal fibroblasts, we performed ultrastructural studies using transmission electron microscopy. Electron micrographs of corneal fibroblasts showed flask-shaped invaginations of the cell membrane and abundant membrane-associated vesicles (Fig 4D, panels 1 and 3). In this cell, caveolae also appeared to undergo fusion to form caveosomes (Fig 4D, panels 2 and 4). These results confirmed the presence of caveolae and caveosomes in human corneal fibroblasts.

To determine whether TGFBIp resides in the caveolae in corneal fibroblasts, we analyzed the distribution of these proteins on ultrathin cryosections. Sections of WT corneal fibroblasts were labeled with anti-TGFBIp. TGFBIp-gold signal (arrow) were found in invaginated structures (caveolae) (arrow head in Fig 4E① and 4E②) on the plasma membrane. TGFBIp-gold signal (arrow) also is distributed in the cytosol (arrow in Fig 4E②) and the lysosomes of WT corneal fibroblasts (circle in Fig 4E③ and 4E④). Caveolae-like structures also appeared in the lysosomes (arrow head in circle of Fig 4E④). Based on these data, we conclude that TGFBIp may reside in the caveolae both at the plasma membrane and inside the cell.

### Internalized TGFBIp is transported to lysosomes for degradation

**Fig 5:**
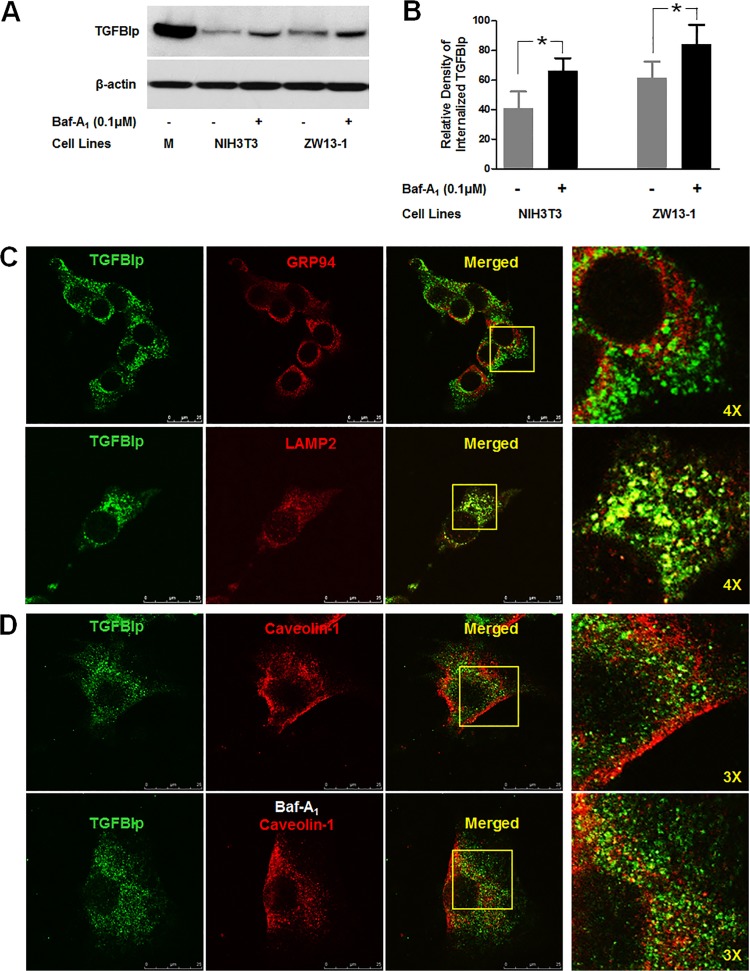
Internalized TGFBIp is transported to lysosomes. **A**. NIH3T3 and ZW13-1 cells were pre-incubated for 60 min in the absence (-) or presence (+) of Baf-A_1_. After incubation, cells were incubated for 120 min in normal media containing TGFBIp and western blotting was performed for TGFBIp. **B**. Densitometric quantitation of the experimental results presented in **A**. A Student’s t-test was performed to determine the significance of differences between treatments with and without Baf-A_1_. Data analysis showed that the p-value was less than 0.05, indicating statistical significance. The experiment was repeated three times independently. **C**. Co-localization of TGFBIp with GRP94, ER marker, and Lamp-2, lysosome marker, in NIH3T3 cells. Cells were subjected to immunocytochemical staining as described in Materials and Methods. Areas of co-localization appear as yellow regions in the merged image. The boxed area in the third panel was magnified and is presented as the fourth panel. **D**. Co-localization of TGFBIp with caveolin-1 in the absence (upper panel) and presence (lower panel) of Baf-A_1_. NIH3T3 cells were grown on glass slides and treated with vehicle or Baf-A_1_ (0.1 μM) for 60 min before incubation for 30 min at 4°C in medium containing ~1 μg/mL TGFBIp. The cells were subjected to immunocytochemical staining as described in Materials and Methods. The boxed area in the third panel was magnified and is presented as the fourth panel.

To determine whether internalized TGFBIp is degraded within the lysosomes, we assessed the level of TGFBIp and its co-localization with lysosomes in the NIH3T3 and ZW13-1 cells treated with Baf-A_1_. We detected a significant increase in TGFBIp in both the cell types after treatment with the inhibitor (Fig 5A and 5B), indicating that TGFBIp is degraded by lysosome-mediated proteolysis or autophagy after internalization. Confocal microscopy revealed that internalized TGFBIp co-localized with the lysosomal marker Lamp-2, but not the ER marker GRP94, in NIH3T3 cells (Fig 5C). Furthermore, increased co-localization of internalized TGFBIp with caveolin-1 was observed in the cells treated with Baf-A_1_ (Fig 5D). These data provide additional supportive evidence that extracellular TGFBIp is internalized by cells and then targeted to the lysosomes for degradation.

### **Interaction of an RGD-motif on TGFBIp with α**_v_ **β**_3_ **integrin mediates TGFBIp entry into cells**

**Fig 6:**
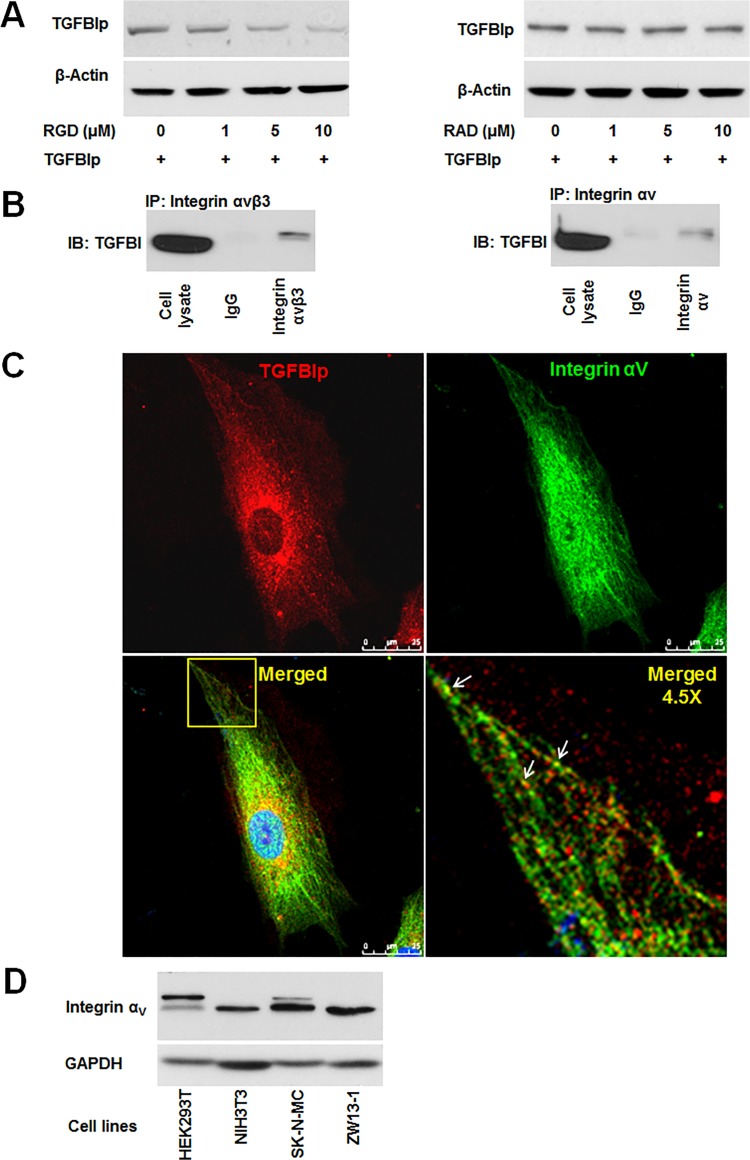
Integrin-dependent endocytosis of TGFBIp in corneal fibroblasts. **A**. Endocytosis of TGFBIp was blocked by RGD peptide in a dose-dependent manner. Corneal fibroblasts were pre-incubated for 30 min in the absence (lane 1) or presence (lanes 2–4) of RGD or RAD peptides. TGFBIp (~1 μg/mL) was added to the medium and the cells were incubated for 120 min at 37°C. TGFBIp levels were measured by western blot analysis. **B**. TGFBIp interacts with integrin α_V_β_3_ and α_V_. Cells were lysed with RIPA buffer and the lysate was immunoprecipitated with anti-integrin α_V_β_3_ (left-hand panel) or anti-integrin α_V_ (right-hand panel) antibody as indicated. Immunoprecipitates were resolved on 10% SDS-PAGE gels and immunoblotted with anti-TGFBIp polyclonal antibody. **C**. Co-localization of integrin α_V_ with TGFBIp was visualized by confocal immunofluorescence microscopy. The merged images show TGFBIp as red, integrin α_V_ as green, and areas of co-localization as yellow. The boxed area in the lower left-hand panel was magnified and is presented as the lower right-hand panel. Arrows identify regions of TGFBIp and integrin α_V_ co-localization. Scale bars, 5 μm. **D**. Western blot analysis of HEK293T, NIH3T3, SK-N-MC, and ZW13-1 cell lines with monoclonal antibody against integrin α_V_. GAPDH was used as a loading control.

Integrins are known to be constitutively endocytosed and recycled [54,55]. Previous studies demonstrated that TGFBIp interacts directly with several integrins, including α_V_β_3_, through mechanisms dependent and independent of the RGD binding motif [56,57]. These data suggest that the RGD motif mediates the internalization of TGFBIp through interaction with integrins. Therefore, we evaluated whether RGD-mediated interactions of TGFBIp with integrins are involved in its internalization. Exogenous human TGFBIp was incubated with corneal fibroblasts in the presence of either RGD peptide or control RAD peptide for 2 h. In the presence of the RGD peptide, the amount of internalized TGFBIp was reduced in a dose-dependent manner (Fig 6A, left panel). However, intracellular TGFBIp levels did not change in cells incubated with the control RAD peptide (Fig 6A, right panel). These results suggest that RGD peptides disrupt TGFBIp internalization by preventing its endocytosis from the ECM.

We also examined the potential association of TGFBIp with α_V_β_3_ integrin by co-immunoprecipitation. Cell lysates from corneal fibroblasts were subjected to immunoprecipitation with specific antibodies against integrin α_V_ or α_V_β_3,_ and the resulting immunoprecipitates were analyzed for the presence of TGFBIp by western blot. Our data demonstrate that TGFBIp was co-immunoprecipitated by both integrin α_V_ and α_V_β_3_ antibodies (Fig 6B), suggesting the association between integrin α_V_β_3_ and TGFBIp. Consistent with these results, confocal microscopy analysis showed the co-localization of internalized TGFBIp and integrin α_V_ in corneal fibroblasts (Fig 6C). Taken together, these data demonstrate that TGFBIp is internalized via the interaction of α_V_β_3_ or/and α_V_β_5_ integrins with an RGD-motif in TGFBIp. The variations in TGFBIp internalization among different cell lines (Fig 2A) may depend on the expression level of integrins as cell surface receptors for TGFBIp. Therefore, we assayed the levels of integrin α_V_ expression in these cell lines. Although the expression of integrin α_V_ showed a different pattern between human (HEK293T and SK-N-MC) and mouse (NIH3T3 and ZW13-1) cell lines, the protein was expressed at a similar level in all the four cell lines (Fig 6D).

### Ubiquitin-mediated proteasome activity is required for internalization of TGFBIp

**Fig 7:**
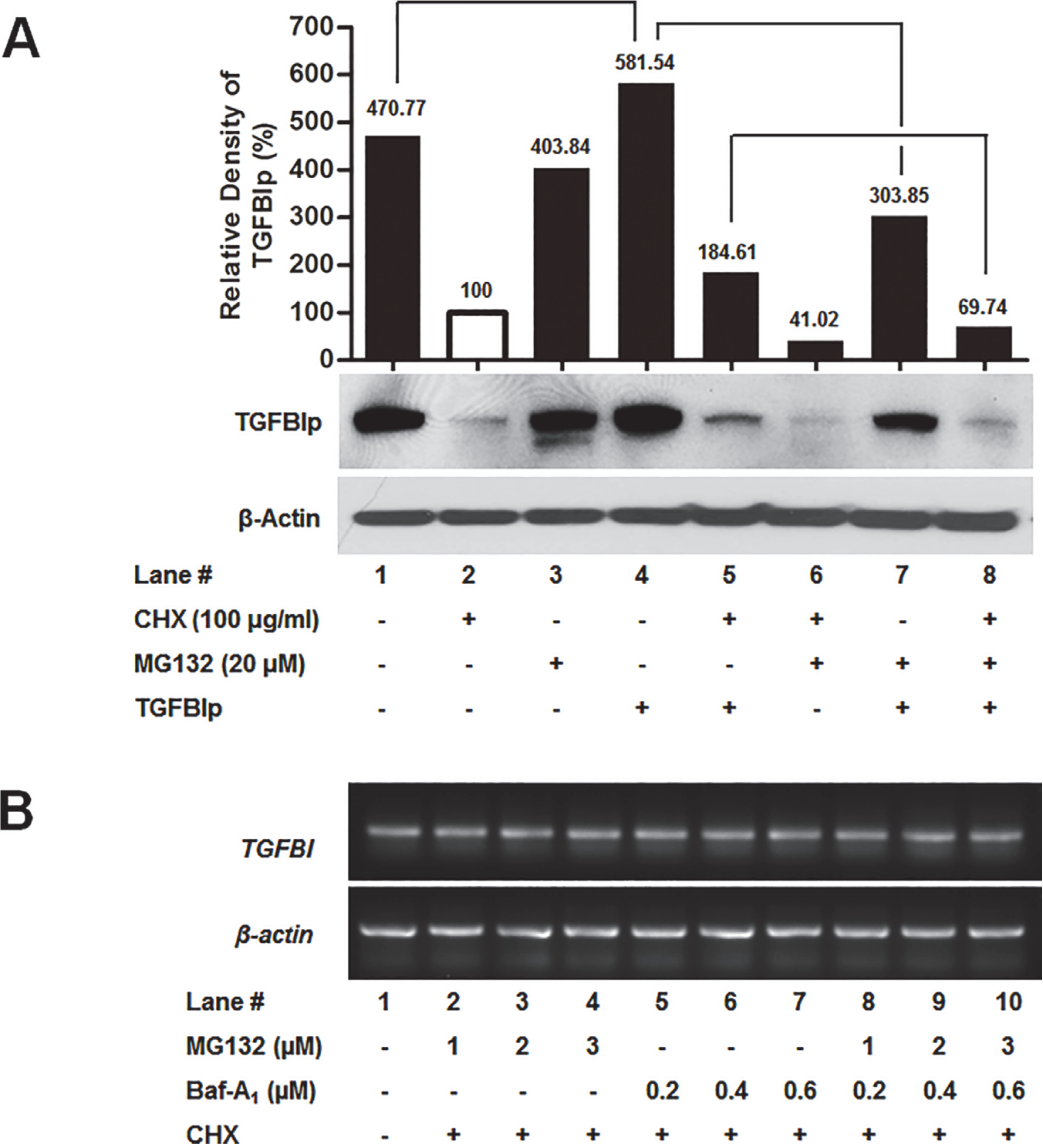
Effects of proteasome inhibitors on TGFBIp internalization. **A**. Corneal fibroblasts were treated with CHX (to inhibit translation), and MG132 (proteasome inhibitors) or Baf-A_1_ (lysosomal inhibitors) for 60 min at 37°C, and then incubated with or without TGFBIp for 120 min at 37°C as indicated. The TGFBIp level was analyzed by western blotting. **B**. RT-PCR analysis of the effect of the specified inhibitors on the mRNA levels of *TGFBI*.

Ubiquitination plays a role not only in proteasome-mediated protein degradation, but also in receptor-mediated endocytosis. One of the non-proteasome functions of ubiquitination is its implication in the process of endocytosis [58]. Therefore, we assessed whether ubiquitination has any effect on TGFBIp endocytosis. To examine the endocytosis of TGFBIp in corneal fibroblasts, we measured TGFBIp internalization after treatment with cycloheximide (CHX), which inhibits translation. As expected, CHX treatment inhibited the expression of TGFBIp (Fig 7A, lane 2). The level of intracellular TGFBIp increased in corneal fibroblasts treated with exogenous TGFBIp, unlike that in the non-treated cells (Fig 7A, lane 4 compared to lane 1). Furthermore, treatment with a chemical inhibitor of the 26S proteasome, MG132, dramatically decreased the level of intracellular TGFBIp after treatment with exogenous TGFBIp (Fig 7A, lane 4 compared to lane 7), suggesting that TGFBIp internalization is facilitated by the ubiquitin-proteasome activity. The addition of MG132 to cells treated with Baf-A_1_ in the presence of CHX further reduced the level of intracellular TGFBIp (Fig 7A, lane 8 compared to lane 5), indicating that ubiquitination is necessary for TGFBIp intracellular trafficking to lysosomes.

To confirm whether changes in the levels of intracellular TGFBIp induced by MG132 and Baf-A_1_ treatments was caused by internalized or newly synthesized TGFBIp, we measured *TGFBI* mRNA expression in cells treated with and without various concentrations of MG132 or/and Baf-A_1_ in the presence of CHX for 120 min by RT-PCR. These inhibitors did not change *TGFBI* mRNA levels (Fig 7B), indicating that MG132 or/and Baf-A_1_ treatments did not regulate *TGFBI* gene levels, but affected the endocytosis pathway of TGFBIp.

## Discussion

Despite the demonstration of the role of TGFBIp accumulation in the pathogenesis of TGFBI-linked corneal dystrophy, the molecular mechanisms involved in extra- and intracellular trafficking of TGFBIp are poorly understood. Cultured corneal fibroblasts from patients with GCD2 exhibit the accumulation of TGFBIp in the lysosomal compartments, suggesting dysfunctional TGFBIp trafficking and turnover.

Current therapeutic strategies for the treatment of TGFBI-linked corneal dystrophy are focused on inhibiting TGFBIp expression by blocking the TGF-β signaling pathway [59]. However, this strategy may affect other critical functions of TGFBIp, such as cell adhesion [60], cell migration [60], and wound healing [10]. Therefore, long-term treatment could result in injury to the cornea in addition to side effects associated with disrupting the crosstalk between TGF-β/Smad and other signaling pathways. Most importantly, blocking TGF-β signaling can result in the dysregulation of cell growth. Therefore, degradation of mutant TGFBIp via internalization before or/and after deposition in the ECM is a potentially effective strategy for targeted drug intervention in TGFBI-linked corneal dystrophy.

In this study, we investigated the trafficking of WT and mutant TGFBIp. Our data demonstrate that TGFBIp is secreted through an ER/Golgi-dependent pathway, and that this secretion is delayed in GCD2 corneal fibroblasts. TGFBIp is internalized via a caveolin-dependent endocytic pathway and transported to the lysosomes for degradation. This internalization is mediated by RGD motif-dependent binding of TGFBIp to integrin α_V_β_3_ or α_V_β_5_. Finally, our results demonstrate that the ubiquitin-proteasome system regulates TGFBIp endocytic trafficking.

TGFBIp was detected in our western blot analysis as two bands (~66 kDa and ~68 kDa). Previous studies have suggested that these bands reflect the absence or presence of a signal peptide, alternative splicing of the *TGFBI* gene [61], or proteolytic processing [62]. In this study, neither band was affected by treatment with BFA nor MON, drugs that disrupt trafficking from the ER to Golgi or from the Golgi to ECM, respectively. Additionally, both bands were detected in samples of conditioned media, although only the ~ 68 kDa band was detected in cells and conditioned media throughout the pulse/chase experiment. These results suggest that the ~66 kDa protein might have been generated by proteolytic cleavage by proteases in the ECM prior to internalization via endocytosis.

Studies have demonstrated that abnormal regulation of ECM components contributes to the progression of many diseases, including fibrosis and cancer [27–29,31]. Therefore, we postulate that the ~66 kDa form of TGFBIp might be important in the progression of TGFBI-linked corneal dystrophy and might facilitate the physiological process related to ECM remodeling, wound healing, development, and cancer. Furthermore, several studies have shown that proteolytic processing of mutant TGFBIp is involved in the pathogenesis of TGFBI-linked corneal dystrophy [62–64]. Recent studies have shown the accumulation of different proteolytic fragments of TGFBIp with diverse cleavage sites in the amyloid deposits of patients with TGFBI-linked corneal dystrophy. Taken together, these results suggest that further proteolysis of this ~66 kDa form by proteases such as matrix metalloproteinases could generate corneal-specific deposition and/or amyloid via distinct aggregation pathways in corneal tissue. Further investigation is required to examine these hypotheses.

Turnover of ECM proteins is an important mechanism for the removal of biologically active proteins from the extracellular environment. Proteins can be degraded in the extracellular space by proteases [23,24] or undergo intracellular degradation in lysosomes or by autophagy after endocytosis [25,26,65,66]. Previous studies have established that the degradation and removal of ECM proteins is involved in development, postnatal tissue remodeling, and tissue repair [5,67]. Accordingly, abnormal degradation and removal of ECM components could contribute to many diseases, including fibrosis, arthritis, and cancer [27–31]. We have previously demonstrated the accumulation of mutant TGFBIp in cytoplasmic or lysosomal compartments due to defective autophagy or delayed fusion between autophagosomes and lysosomes in GCD2 corneal fibroblasts [33]. Therefore, delayed fusion between lysosomal compartments and internalized caveolae and/or caveosome vesicles containing TGFBIp should be the subject of future studies.

Our data using immune-electron microscopy, confocal microscopy, and pharmacological inhibitors demonstrate that TGFBIp may be internalized via caveolae-mediated endocytic pathways. Furthermore, Cav-1–null cells failed to show internalization of TGFBIp. Caveolae and caveosome vesicles are abundant in corneal fibroblasts, indicating an active caveolae-mediated endocytosis in these cells. It is interesting to speculate on the possibility that the internalization of mutant TGFBIp could be inhibited in GCD2 corneal fibroblasts by mechanisms involving the polymerization of mutant TGFBIp and age-related failure of endocytosis [68]. Levels of caveolin protein are upregulated in an age-dependent manner in senescent fibroblasts and aged animal tissues [69,70]. This might explain the age-dependent accumulation of mutant TGFBIp in corneal stroma. However, in our study, the expression levels of caveolin-1 and -2 did not differ significantly between WT and GCD2 cells. These findings point to an important therapeutic application for TGFBI-linked corneal dystrophy, because mutant TGFBIp could be removed by endocytosis by corneal fibroblasts in the corneal stroma. Our data demonstrate that TGFBIp co-localized with integrins in the intracellular vesicles in corneal fibroblasts. Moreover, we showed that TGFBIp interacted with α_V_ integrin, and that RGD peptide inhibited TGFBIp internalization in a dose-dependent manner. These data suggest that the RGD motif could mediate interaction with integrins, even though TGFBIp is also able to interact with integrins in the absence of this motif [71]. Although the internalization of most integrins occurs via a clathrin-mediated process [72,73], caveolin-1 has been shown to regulate the endocytosis of fibronectin-binding β_1_ integrins [74]. Our data indicate that TGFBIp internalization may depend on the expression of integrin or/and caveolins. However, HEK293T cell did not internalize TGFBIp even they do express avb3 integrin. “It has been demonstrated that integrins, α5β1, α8β1, αIIbβ3, αVβ1, αVβ3, αVβ5, αVβ6, and αVβ8, can recognize RGD motifs in their ligands [75]. These data reveal that TGFBIp could interact with these integrins, and may be internalized via caveolae or/and clathrin-mediated endocytosis or/and other pathways. The internalization of many integrin heterodimers occurs by clathrin‑dependent, clathrin‑independent, and caveolin‑dependent endocytic mechanisms. Integrin heterodimers can follow more than one internalization route. For example, α5β1 integrin can be internalized by both clathrin‑dependent [76] and caveolin‑dependent endocytic mechanisms [74]; αvβ3 integrin is recruited to caveolae and internalized in caveolin 1-dependent manner [77] and can also enter the cell via clathrin‑coated pit structures [78]. Furthermore, recent advances in understanding of integrin endocytosis have elucidated the molecular connection between the integrin and endocytic machineries, such as adaptors and associated proteins [79]. In addition, pathways of integrin internalization are cell type-dependent and rely on cellular processes, such as migration *etc*. [79]. Moreover, several integrins, αvβ3 [77], α5β1 [74], and αLβ2 [80], are internalized via caveolin‑dependent endocytosis [77]. Given these data, although we cannot rule out that TGFBIp is also internalized via clathrin‑dependent and other endocytic mechanisms, at the very least our data indicate that integrin αVβ3 may mediate the internalization of TGFBIp via a caveolin-dependent pathway. Further, this data indicate that TGFBIp endocytosis might be depending on the expression of caveolin-1 and -2 rather than the expressions of integrin avb3 or other integrins. This possibility is further supported by the differences in the rate of TGFBIp internalization among HEK293T, NIH3T3, SK-N-MC, and ZW13-1 cell lines. We showed that caveolin-1 and -2 were expressed differentially in NIH3T3, SK-N-MC, and ZW13-1 cell lines, but were not expressed in the HEK293T cell line. Additionally, HEK293T cells express higher levels of α_V_, α_5_, and β_1_ integrin, but the expression of α_V_β_3_ and α_V_β_5_ integrin is undetectable [81,82]. These data suggest that HEK293T cells do not possess caveolae-dependent endocytic pathways for TGFBIp, which in turn might have contributed to the inability to detect TGFBIp internalization in these cells. However, we cannot rule out a very low rate of TGFBIp internalization in HEK293T cells. Taken together, our findings indicate that the expression of caveolins and α_V_β_3_ integrin may play a significant role in the degradation of TGFBIp via endocytosis. This notion is supported by previous evidence that TGFBIp function is directly linked to integrin signaling [13].

The ubiquitin-proteasome system regulates receptor-mediated endocytosis through the ubiquitination of the internalized receptor [83,84]. The reduction in TGFBIp levels after MG-132 treatment indicates that TGFBIp internalization is regulated by ubiquitin-proteasome activity. Accordingly, the reduced level of TGFBIp in the presence of MG132 may be due to the inhibition of endocytosis rather than increased degradation. We postulate that the reduced activity of the ubiquitin-proteasome system could induce the accumulation of mutant TGFBIp in the ECM by inhibiting internalization. Age-dependent TGFBIp accumulation and deposition has been observed in the corneal stroma of patients with TGFBI-linked corneal dystrophy. The ubiquitin-proteasome system exhibits a gradual decrease with age [85] in a variety of tissues, including the eye [85–89]. Furthermore, we have previously shown that the levels of polyubiquitinated proteins are higher in GCD2 corneal fibroblasts than in the WT cells, indicating the loss of proteasome activity in GCD2 corneal fibroblasts [33]. Taken together, these results support the hypothesis that impaired TGFBIp endocytosis resulting from an age-dependent decrease in the ubiquitin-proteasome system leads to age-dependent accumulation and deposition of TGFBIp in TGFBI-linked corneal dystrophy. However, further studies are required to understand the mechanism by which accumulation and aggregation of mutant TGFBIp is associated with the ubiquitin-proteasome system.

## Conclusion

Our data suggest that a disturbance in the intracellular trafficking of mutant TGFBIp can lead to TGFBIp-linked corneal dystrophies. Furthermore, it seems reasonable to hypothesize that altered ECM turnover, which is regulated by endocytosis, can trigger the aggregation, deposition, and accumulation of mutant TGFBIp in the corneal stroma. A better understanding of the molecular events involved in the intracellular and extracellular trafficking of ECM proteins such as TGFBIp will allow us to better address this phenomenon. Such knowledge might lead to the identification of novel targets and the development of new therapies for the treatment of TGFBI-linked corneal dystrophy.

## Supporting Information

S1 Table The PCR primer pairs used for RT-PCR. (DOCX)

S1 Fig *CAV-1* and *TGFBI* gene expressions in HEK293, WT corneal fibroblasts, and Cav1 null cells (CAV-1 KO). Amplified DNA visualized by ethidium bromide staining on a 1.2% TAE agarose gel.
(TIF)
