# Supplementary material for: Lysosomal Trafficking of TGFBIp via Caveolae-Mediated Endocytosis
Source: PLoS One. 2015 Apr 8;10(4):e0119561. doi: 10.1371/journal.pone.0119561 (PMC4390356; doi:10.1371/journal.pone.0119561)
Supplement: S1 Table — (DOCX) [file pone.0119561.s001.docx]

**Table S1. The PCR primer pairs used for RT-PCR**

| **Genes** | **Accession**  **number** | **Forward primers** | **Reverse primers** | | **Product**  **Size (bp)** | | |
| --- | --- | --- | --- | --- | --- | --- | --- |
| *CAV1* | NM_001753.4 | 5’-AGCTGGTCAAGATTGACTTTGAA -3’ | | 5’-TGCACTGAATCTCAATCAGGAAG -3’ | | 246 |  |
| *TGFBI* | NM_000358 | 5’-GTGTGTGCTGTGCAGAAGGT-3’ | | 5’-TTGAGAGTGGTAGGGCTGCT-3’ | 172 | | |
| *β-Actin* | NM_001101 | 5’-GGACTTCGAGCAAGAGATGG-3’ | | 5’-AGCACTGTGTTGGCGTACAG-3’ | 234 | | |
