# Supplementary figures and images for: Lysosomal Trafficking of TGFBIp via Caveolae-Mediated Endocytosis
Source: PLoS One. 2015 Apr 8;10(4):e0119561. doi: 10.1371/journal.pone.0119561 (PMC4390356; doi:10.1371/journal.pone.0119561)

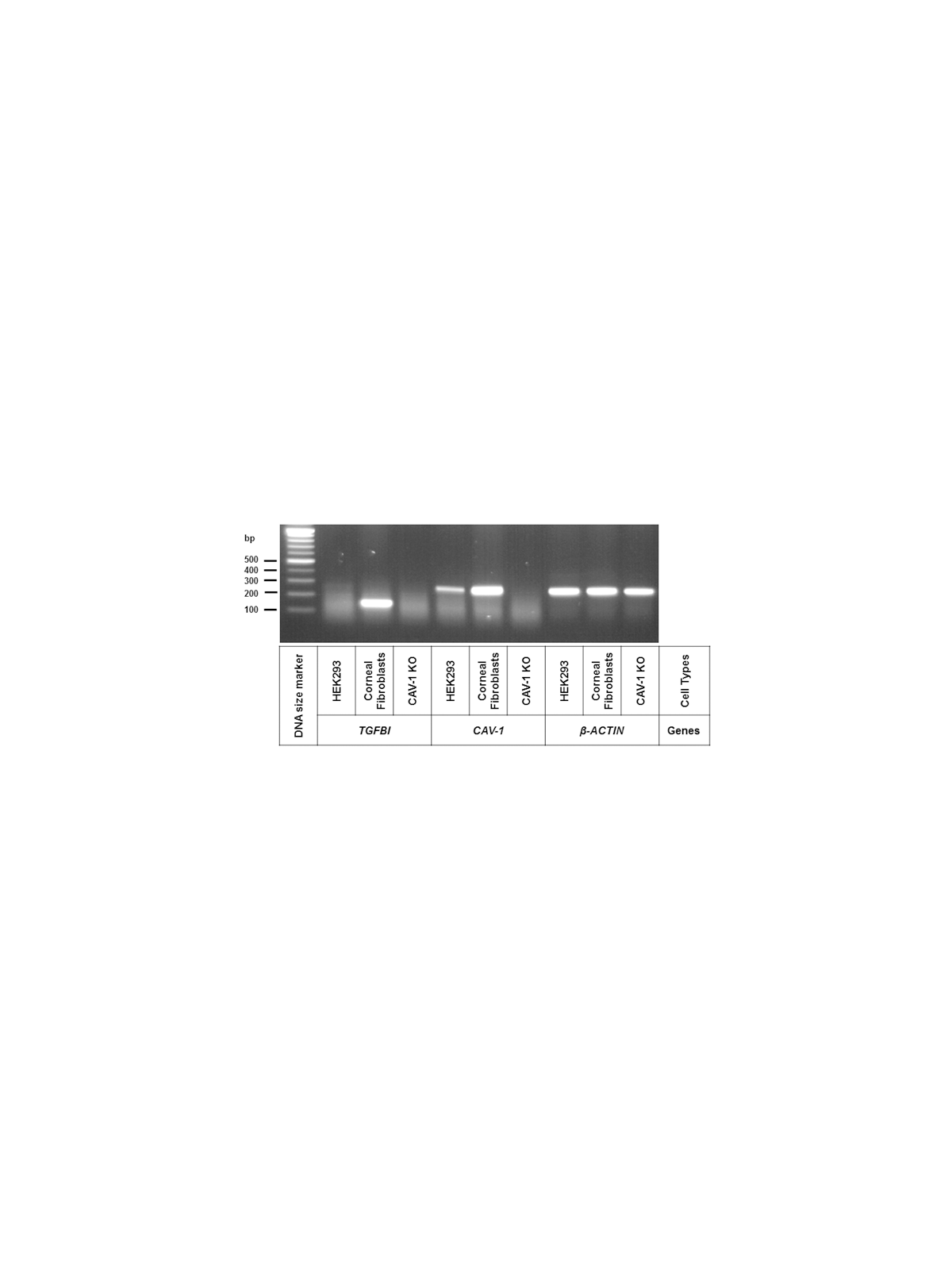

Supplement: S1 Fig — Amplified DNA visualized by ethidium bromide staining on a 1.2% TAE agarose gel. (TIF) [file pone.0119561.s002.tif]
